# A role for fat precursors in the marrow

**DOI:** 10.7554/eLife.58084

**Published:** 2020-05-13

**Authors:** Noriaki Ono

**Affiliations:** School of Dentistry, University of MichiganAnn ArborUnited States

**Keywords:** adipocyte, bone marrow mesenchymal stem cells, osteoblast, blood vessel, single cell RNA-seq, Mouse

## Abstract

A group of cells that can become adipocytes controls the formation of blood vessels in the bone marrow, and also regulates the differentiation of resident mesenchymal progenitor cells.

**Related research article** Zhong L, Yao L, Tower RJ, Wei Y, Miao Z, Park J, Shrestha R, Wang L, Yu W, Holdreith N, Huang X, Zhang Y, Tong W, Gong Y, Ahn J, Susztak K, Dyment N, Li M, Long F, Chen C, Seale P, Qin L. 2020. Single cell transcriptomics identifies a unique adipose lineage cell population that regulates bone marrow environment. *eLife* **9**:e54695. doi: 10.7554/eLife.54695

Forget your prejudices against adipocytes – fat cells have important roles to play in the body. This is particularly the case in the bone marrow, where bone and blood are constantly renewed throughout life. The bone marrow provides an interface between bones and the blood – blood cells constantly come and go, while bone cells are long-term inhabitants of the marrow space. Bone cells are derived from so-called bone marrow stromal cells (BMSCs), which are usually found near blood vessels. RNA-seq experiments show that a substantial group of BMSCs express genes normally associated with fat cells (Baccin et al., 2020; Baryawno et al., 2019; Tikhonova et al., 2019; Wolock et al., 2019). Moreover, older bones have an increased amount of fat in their marrow, and a large number of BMSCs express the receptor for leptin, a hormone that is often found in fat cells (Zhou et al., 2014). Yet, the roles that fat cells and their precursors play in the bone marrow environment remain largely unknown.

Now, in eLife, Ling Qin from the University of Pennsylvania and co-workers – including Leilei Zhong as first author – report the discovery of a group of cells in the bone marrow that they have named marrow adipogenic lineage precursor (MALP) cells (Zhong et al., 2020).

First, Zhong et al. performed single-cell RNA-seq on BMSCs from young, adult, and aging mice. They fluorescently labeled these cells (Ono et al., 2014) and then used a computational approach to define several distinct stages of mesenchymal progenitors in their paths to becoming bone cells – osteoblasts – and adipocytes. In particular they discovered the MALP cells, which can become fat cells and increase in number as the mice age. These cells express many genes associated with adipocytes, including adiponectin, but do not yet accumulate lipids.

Strikingly, MALP cells form a vast three-dimensional network surrounding blood vessels in the bone marrow known as sinusoidal vessels. MALP cells express a gene called *Adipoq*, which is also expressed by subcutaneous fat cells. When cells expressing *Adipoq* were selectively removed from mice, the sinusoidal vessels were severely disrupted. Moreover, loss of these cells caused a massive increase in bone trabeculae (thin rods and plates of bone tissue) throughout marrow space.

However, it has recently been shown that factors called adipokines, including adiponectin and leptin, which are produced by subcutaneous fat cells, negatively regulate bone formation (Zou et al., 2019). This means that the changes observed in the bone marrow when cells expressing *Adipoq* are removed could simply be due to the disappearance of subcutaneous fat cells. To test whether this was the case, Zhong et al. transplanted subcutaneous fat into mice and then removed the host cells that expressed *Adipoq*﻿. The mice now had subcutaneous fat cells, but no MALP cells, and still exhibited disrupted sinusoidal vessels and excess trabeculae. This result demonstrates that the changes observed in the bone marrow upon the removal of cells expressing *Adipoq* are specifically caused by MALP cell removal. These findings suggest that MALP cells are locally important components of the bone marrow environment, particularly in regulating marrow vasculature and bone formation, perhaps by secreting cytokines (Figure 1).

**Figure 1.**
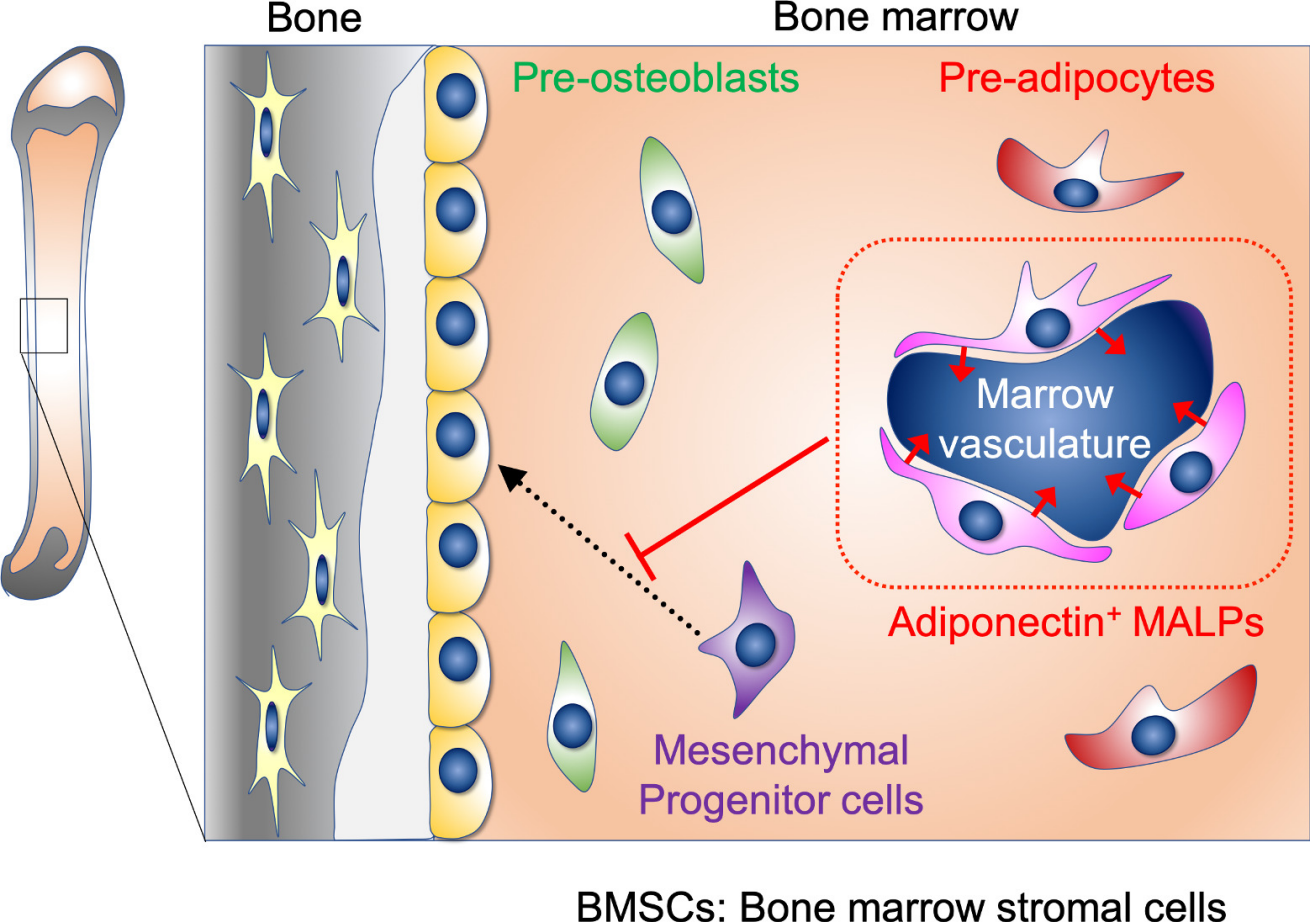
MALP cells and the formation of bone and blood vessels in the bone marrow (Zhong et al., 2020). Schematic view of bone (grey) and bone marrow (orange). Bone marrow contains many different types of mesenchymal stromal cells: these include mesenchymal progenitor cells (purple), pre-osteoblasts (green), and pre-adipocytes (red). Mesenchymal progenitor cells and pre-osteoblasts can both give rise to the osteoblasts that line the bone surface (smooth yellow cells) and to the osteocytes that are embedded within the bone (jagged yellow cells). A MALP cell is type of pre-adipocyte, and MALP cells that express a hormone called adiponectin have important roles in: i) maintaining the marrow vasculature (arrow-ended red lines); ii) preventing mesenchymal progenitor cells from differentiating into osteoblasts (flat-ended red lines).

Together with a recent study that demonstrates the importance of pre-adipocyte-like cells in bone regeneration (Matsushita et al., 2020), these findings establish the concept that marrow adipocyte precursors (including MALP cells) play active roles in bone physiology and regeneration. The precise nature of MALP cells still requires further definition. For instance, many types of adipocyte-related cells have been described to date in the bone marrow. Do these cells represent separate or overlapping entities? Do they occupy different locations in bone marrow? If so, does that mean they have different roles? Another important question is whether there is any special feature that determines the identity of marrow adipocytes. How are they different from adipocytes in other areas of the body, and how do they differ metabolically? It is intriguing to think that the adipocyte precursor identity may confer cells with some metabolic advantages to secrete large amounts of cytokines.
